# High-efficiency generation of induced pluripotent mesenchymal stem cells from human dermal fibroblasts using recombinant proteins

**DOI:** 10.1186/s13287-016-0358-4

**Published:** 2016-07-30

**Authors:** Fanfan Chen, Guoqiang Zhang, Ling Yu, Yanye Feng, Xianghui Li, Zhijun Zhang, Yongting Wang, Dapeng Sun, Sriharsa Pradhan

**Affiliations:** 1 Shanghai R&D Center, New England Biolabs, Shanghai, 201203 China; 2 New England Biolabs, Ipswich, MA 01938 USA; 3 Med-X Research Institute of Shanghai Jiao Tong University, Shanghai, 200030 China

**Keywords:** Induced pluripotent stem cell, Induced pluripotent mesenchymal stem cell, Inactivated viral particle, In-vitro differentiation, Whole genome bisulfite sequencing

## Abstract

**Background:**

Induced pluripotent mesenchymal stem cells (iPMSCs) are novel candidates for drug screening, regenerative medicine, and cell therapy. However, introduction of transcription factor encoding genes for induced pluripotent stem cell (iPSC) generation which could be used to generate mesenchymal stem cells is accompanied by the risk of insertional mutations in the target cell genome.

**Methods:**

We demonstrate a novel method using an inactivated viral particle to package and deliver four purified recombinant Yamanaka transcription factors (Sox2, Oct4, Klf4, and c-Myc) resulting in reprogramming of human primary fibroblasts. Whole genome bisulfite sequencing was used to analyze genome-wide CpG methylation of human iPMSCs. Western blot, quantitative PCR, immunofluorescence, and in-vitro differentiation were used to assess the pluripotency of iPMSCs.

**Results:**

The resulting reprogrammed fibroblasts show high-level expression of stem cell markers. The human fibroblast-derived iPMSC genome showed gains in DNA methylation in low to medium methylated regions and concurrent loss of methylation in previously hypermethylated regions. Most of the differentially methylated regions are close to transcription start sites and many of these genes are pluripotent pathway associated. We found that DNA methylation of these genes is regulated by the four iPSC transcription factors, which functions as an epigenetic switch during somatic reprogramming as reported previously. These iPMSCs successfully differentiate into three embryonic germ layer cells, both in vitro and in vivo. Following multipotency induction in our study, the delivered transcription factors were degraded, leading to an improved efficiency of subsequent programmed differentiation.

**Conclusion:**

Recombinant transcription factor based reprogramming and derivatization of iPMSC offers a novel high-efficiency approach for regenerative medicine from patient-derived cells.

**Electronic supplementary material:**

The online version of this article (doi:10.1186/s13287-016-0358-4) contains supplementary material, which is available to authorized users.

## Background

Induced pluripotent stem cells (iPSCs) are somatic cells whose characteristics are reprogrammed by ectopic expression or by introduction of defined transcription factors to resemble those of embryonic stem cells (ESCs) [1]. Yamanaka and colleagues [1–4] generated the first iPSCs using ectopic expression of a series of transcription factors, including Oct4, Sox2, Klf4, and c-Myc. Following their success, many types of mammalian cells have been successfully induced to the pluripotent state by related methods. These cells are thought to hold promise for regenerative medicine, drug screening, disease modeling, and basic research [1–4]. However, the reprogramming mechanism is unclear and some problems remain unresolved, including safety issues stemming from the use of viral vectors, especially in light of the oncogenic potential of two of the key transcription factors, c-Myc and Klf4 [5]. Additionally, any insertion of foreign DNA into the genome poses the risk of insertional mutagenesis. Integrated vectors are therefore not optimal reprogramming agents due to safety concerns.

In an attempt to circumvent insertional mutagenesis, significant efforts have been put into the reprogramming technologies that avoid ectopic DNA integration. Until now integration-free iPSCs have been derived through expression of reprogramming factors (RFs) using excisable (transposon and floxed lentivirus) or nonintegrating vectors (plasmid, episomal, and adenovirus vector) [6–9]. However, iPSCs generated by these approaches either are left with a short vector DNA after excision or are not entirely free of the risk of transgene insertions [6–9].

One possible way to avoid introducing exogenous genetic modifications to target cells is to deliver the reprogramming proteins directly into cells, rather than relying on the transcription from delivered genes. Zhou et al. [9] reported the generation of mouse iPSCs using four rounds of protein transduction of the reprogramming factors tagged with polyarginine in the presence of VPA. They found that the transduced proteins appeared to be stable inside the cells for up to 48 h. This study first demonstrated that somatic cells could be fully reprogrammed into pluripotent mesenchymal stem cells (MSCs) by direct delivery of recombinant reprogramming proteins using a protein transduction signal peptide. Human iPSC lines have also been established with protein transductions without VPA [10]. However, this method is extremely inefficient (0.001 %), and slow (8 weeks). This low efficacy might partially be a result that investigators used whole cell lysates rather than purified proteins as a source of transcription factors. Many other groups have now introduced TAT-mediated proteins into mouse and human somatic cells for reprogramming purposes [11–14].

iPSCs have the ability to undergo self-renewal and have the potential to differentiate into every cell type of the human body and represent a potential source of renewable cells for cell therapies and regenerative medicine. Pluripotent stem cells can further give rise to progenitor cells, such as MSCs that can differentiate into mesodermal derivatives like bone, fat, cartilage, tendon, and muscle. Here we have developed a novel recombinant protein transduction method to generate human iPMSCs from patient-derived fibroblast using a replication-defective and persistent Sendai virus (SeVdp) vehicle, Hemagglutinating Virus of Japan Envelope (HVJ-E). We have delivered four recombinant purified transcription factors (Oct4, Sox2, Klf4, and c-Myc) into human fibroblasts and obtained reprogrammed iPMSCs with higher efficiency than the methods reported previously. The human iPMSC genomes were analyzed for DNA methylation changes and monitored for iPMSC and iPSC specific cell surface markers. The putative iPMSCs were further differentiated into osteoblast, adipocyte, pancreatic islet, neural, and teratoma cells.

## Methods

### Expression and purification of Oct4, Sox2, c-Myc, and Klf-4 protein factors

*Oct4*, *Sox2*, and *c-Myc* transcription factors were cloned into pET28a. *Klf-4* was cloned into mammalian expression vector pcDNA 3.1. Fusion protein constructs in a pET28a background were transformed into Rosetta DE3 and selected on a LB agar with kanamycin (100 mg/l) plate at 37 °C overnight. The colonies were inoculated in 100 ml of LB-kanamycin and grown at 37 °C overnight. For expression, 10 ml of the overnight culture was inoculated into 1 l LB-kanamycin at 37 °C for 2–3 h until OD_600_ reached 0.6–0.8. IPTG was added to a final concentration of 0.5 mM, and the culture was incubated for another 16 h at 18 °C. Cells were harvested and stored at –20 °C. Unless otherwise indicated, all subsequent steps were performed at 4 °C. The cell pellet was suspended at 1:20 dilution on ice in buffer containing 20 mM Tris–Cl pH 8.5, 1 M NaCl, 1 mM EDTA, 0.1 mM PMSF, and 5 % glycerol. This suspension was sonicated at ~36 W, at 40-min intervals for 3 min until >90 % of the cells were broken. The cell lysate was centrifuged for 30 min at 8000 rpm to sediment cellular debris. The pellet was suspended at 1:20 dilution on ice in buffer containing 20 mM Tris–Cl, pH 8.5, 1 M NaCl, 8 M urea, 20 mM β-ME, and 20 mM imidazole at room temperature and gently stirred overnight. The suspended pellet was centrifuged at 18,000 rpm for 1 h at 12 °C and supernatant collected. The supernatant was loaded onto a 5-ml nickel column under denaturing conditions (buffer A: 20 mM Tris–Cl, pH 8.5, 1 M NaCl, 8 M urea, and 20 mM imidazole). Unbounded protein was washed with 20 column volumes of buffer A, and the bound protein was eluted with buffer B (20 mM Tris–Cl, pH 8.5, 1 M NaCl, 8 M urea, and 500 mM imidazole). DTT was added to the elution fractions to a final concentration of 5 mM followed by gentle stirring at 4 °C for 2–4 h.

For Klf-4 purification, pcDNA3.1-Klf-4 construct was transfected into FreeStyle™ 293-F cells in a spinner flask and cells were incubated on an orbital shaker platform at 125 rpm in a 37 °C incubator with humidity and 8 % CO_2_ for 48 h. Then 200 ml of transfected 293F cells were harvested and resuspended in 200 ml lysis buffer (50 mM Tris–Cl, pH 7.3, 150 mM NaCl, 1 % CA-630, aprotinin 1 μg/ml, leupetin 1 μg/ml, pepstatin 1 μg/ml, bestatin 1 μM, and 1 mM PMSF) and shaken on ice for 30 min. The cell lysate was centrifuged for 40 min at 14,000 rpm to sediment cellular debris. The supernatant was filtered through a 0.22 μM membrane and loaded onto a 5-ml DEAE column and the flow through was collected. This flow-through protein solution was loaded onto a 1-ml nickel column, washed with 40 mM imidazole and then eluted by elution buffer (50 mM Tris–Cl, pH 7.3, 150 mM NaCl, and 250 mM imidazole) with 50 column volumes in a 0–100 % gradient. Purified Klf4 was dialyzed into the storage buffer (20 mM Tris–Cl pH 8, 1 mM DTT, 100 mM NaCl, and 50 % glycerol) and stored at –80 °C.

### Refolding of proteins and protein binding assay

The denatured Oct4, Sox2, and c-Myc eluate was diluted ~10-fold into pre-cooled refolding buffer (50 mM Tris–Cl, pH 8.5, 500 mM NaCl, 500 mM Arg, 0.1 % PEG4000, 0.1 mM EDTA, 1 mM GSH, and 0.1 mM GSSH) with gentle stirring to a final concentration of proteins between 0.05 and 0.1 mg/ml and was incubated at 4 °C for 48 h. The refolded protein was dialyzed against continuous exchange of fresh dialysis buffer (1 × PBS buffer, 5 mM β-ME, and 5 % glycerol) at 4 °C for 64 h. Refolded proteins were loaded onto a 1-ml nickel column and the target protein eluted with 100 % buffer B (buffer A: 1 × PBS buffer, 5 mM β-ME, 5 % glycerol, and 10 mM imidazole; buffer B: 1 × PBS buffer, 5 mM β-ME, 5 % glycerol, and 500 mM imidazole) in a 50 ml gradient. The refolded proteins were dialyzed against the storage buffer (1 × PBS buffer, 5 mM DTT, and 50 % glycerol) and stored at –80 °C. Oct4, Sox2, c-Myc, and Klf-4 were further tested for exonuclease, endonuclease, and endotoxin presence. DNA-binding activity of these protein factors was tested with electrophoretic mobility shift assays (EMSAs) using oligonucleotide duplexes representing the following sequences: Sox2, GAGACTTAATAACAAAGACCTGAAGCAGAGTCAG; Oct4, CTCGAGACTTAATAATTTGCATACCCTGAAGGCAGGAGTCAG; c-Myc, CTCGAGACTTAATACACGTGACCTGAAGGCAGAGTCAG; and Klf-4, CTGACTCTGCCTTCAGGTCACCCTATTAAGTCTCGAG.

The linear DNA fragments (100 nM) were incubated at 95 °C for 5 min in 1 × annealing buffer (10 mM Tris–Cl, pH 8.0, 50 mM NaCl, and 1 mM EDTA) and gradually cooled down to room temperature for annealing. For DNA binding reaction, 10 nM of the annealed oligonucleotides were incubated with the target proteins (2 μg) in 20 μl binding buffer (10 mM Hepes, pH 7.8, 5 mM MgCl_2_, 50 mM KCl, 0.5 mM DTT, and 1 % glycerol) at 26 °C for 1 h. Electrophoretic mobility assay was performed by adding 2 μl of 10 × gel loading dye (50 % glycerol and 10 % SYBR green dye) to the DNA protein mixture and 10 μl of each sample was analyzed using a native polyacrylamide gel (4 % acrylamide, 2.5 % glycerol, and 0.5 × TBE). The samples were resolved at 4 °C.

### Generation of putative induced stem cells using HVJ envelope

**Fig. 1:**
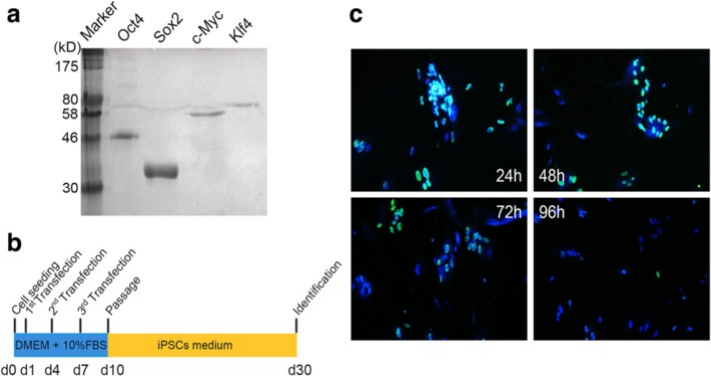
Characterization of recombinant Yamanaka transcription factors for transduction. **a** Recombinant refolded and purified Oct4, Sox2, c-Myc, and Klf4 were analyzed using denaturing Bis-Tris NuPage gel. Marker protein standards are shown in lane 1. **b** Schematic protocol depicting the process and timeline for generating iPMSCs. **c** Protein transduction and visualization in human fibroblasts 24, 48, 72, and 96 h post HVJ-E transduction of cells. Cells were stained with Hochest 33342 dye for nuclei (*blue*), and immunostained with anti-His antibody (*green*) for localization of the recombinant Yamanaka transcription factors. Merged images are shown for 24, 48, 72 and 96 h post transduction. *d* day, *IPSC* induced pluripotent stem cell

The HVJ envelope (HVJ-E) transfection kit (GenomONE™) was purchased from Cosmo Bio Co., Ltd. Inactivation of HVJ has been confirmed for each lot by the viral proliferative potential rule-out test, using cultured cells and fertilized chicken eggs. Human fibroblasts were seeded at 2 × 10^5^ cells per well in normal culture media (DMEM supplemented with 10 % FBS). On the next day, media were changed to fresh media supplemented with the HVJ-E transduction complex. After overnight culture, the protein transduction media were replaced by normal culture media, and cells were cultured for an additional 48 h before repeating the same protein transduction cycle. After three rounds of protein transduction, cells were passaged onto VTN-coated dishes at day 10 in iPSC culture media (Essential 8™ Medium; Invitrogen). Media were changed every 3–4 days and cultured for another 20 days (Fig. 1b).

### Localization of transfected reprogramming proteins

To determine the intracellular localization of transcription factors, protein-transduced cells were immunostained and examined by fluorescent microscopy. Cells were harvested 24, 48, 72 and 96 h post transduction, washed with PBS, and fixed in 4 % (v/v) formaldehyde in PBS at room temperature for 1 min. Cells were incubated with the diluted primary anti-his tag antibody (Novagen) at 1:1000 dilution at room temperature for 1 h. After washing three times with PBS, fluorescence-conjugated secondary antibody (anti-mouse IgG (H + L) Alexa Fluor® 488 Conjugate; CST) at 1:1000 dilution was added and incubated for 1 h at room temperature in the dark. Cells were again washed with PBS and nuclear DNA was stained by 1:3000 diluted Hoechst 33342 stain (Sigma-Aldrich). Images were captured by Olympus DP70 microscope.

### Surface antigen analysis of putative induced stem cells

Cell surface antigens for human iPSCs were analyzed with FACS. Cells (1 × 10^5^ cells per well) were incubated with one of the following primary antibodies: anti-CD24 antibody (Abcam), 1:100 dilution; anti-SSEA3 antibody (Abcam), 1:100 dilution; anti-CD105 antibody (Abcam), 1:200 dilution; anti-Nanog antibody (CST), 1:100 dilution; anti-Sox2 antibody (CST), 1:300 dilution; and anti-Oct4 antibody, 1:600 dilution (CST). After 1 h of incubation at 4 °C, the cells were washed, centrifuged, stained with 100 μl of a secondary antibody selected from anti-mouse IgG (H + L) Alexa Fluor® 488 Conjugate (CST) at 1:1000 dilution, anti-rat IgG (H + L) Alexa Fluor® 488 Conjugate (CST) at 1:1000 dilution, and anti-rabbit IgG (H + L) Alexa Fluor® 488 Conjugate (CST) at 1:1000 dilution. The stained cell pellets were suspended in 200 μl PBS/10 % FCS at 4 °C for flow cytometry analysis. All antibodies were validated for antigen specificity.

### Immunofluorescence assays of putative induced stem cells and quantitative PCR

ALP staining was performed using the Alkaline Phosphatase Detection Kit (Millipore) as instructed by the manufacturer. Immunocytochemistry was performed using standard protocols. Briefly, cells were fixed using 4 % paraformaldehyde (PFA; Sigma-Aldrich), washed three times with PBS, and then incubated in PBS containing 0.3 % TritonX-100 (Sigma-Aldrich) and 5 % BSA for 1 h at room temperature. The cells were then incubated with primary antibody at 4 °C overnight: anti-CD24 antibody (Abcam), 1:100 dilution; anti-SSEA3 antibody (Abcam), 1:100 dilution; anti-CD105 antibody (Abcam), 1:200 dilution; anti-Tra1-60 (Millipore), 1:200 dilution; anti-Tra1-81 (Millipore), 1:200 dilution; anti-Nanog antibody (CST), 1:100 dilution; anti-Sox2 antibody (CST), 1:300 dilution; and anti-Oct4 antibody (CST), 1:600 dilution.

After washing three times with PBS, cells were incubated with secondary antibodies: anti-mouse IgG (H + L) Alexa Fluor® 488 conjugate (CST), 1:1000 dilution; anti-rat IgG (H + L) Alexa Fluor® 488 conjugate (CST), 1:1000 dilution; and anti-rabbit IgG (H + L) Alexa Fluor® 488 conjugate (CST), 1:1000 dilution. Nuclei were detected by Hoechst 33342 (Sigma-Aldrich) staining. Images were captured using an Olympus DP70 digital camera. Isotype controls are shown in Additional file 1: Figure S1.

Total RNA was extracted using TRIZol® Reagent (Life Technologies) followed by cDNA synthesis using M-MuLV Reverse Transcriptase and Oligo (dT)23VN (NEB). Quantitative PCR (qPCR) was performed with iTaq™ Universal SYBR® Green Supermix (Bio-Rad) using the primers presented in Table 1 and RT-PCR was performed for gene expression studies using the primers presented in Table 2. All antibodies were validated for antigen specificity.

**Table 1:** Primers for quantitative PCR reactions

Gene name	Forward primer	Reverse primer
*β-actin*	CATGTACGTTGCTATCCAGGC	CTCCTTAATGTCACGCACGAT
*Klf4*	CCCACATGAAGCGACTTCCC	CAGGTCCAGGAGATCGTTGAA
*Nanog*	TTTGTGGGCCTGAAGAAAACT	AGGGCTGTCCTGAATAAGCAG
*c-Myc*	GGCTCCTGGCAAAAGGTCA	CTGCGTAGTTGTGCTGATGT
*Sox2*	GCCGAGTGGAAACTTTTGTCG	GGCAGCGTGTACTTATCCTTCT
*Oct4*	CTGGGTTGATCCTCGGACCT	CCATCGGAGTTGCTCTCCA
*ABCG2*	CAGGTGGAGGCAAATCTTCGT	ACCCTGTTAATCCGTTCGTTTT
*hTERT*	TAATGGGCTCCTTTCACCTG	CAGTGCGTCTTGAGGAGCA
*Insulin*	GCAGCCTTTGTGAACCAACAC	CCCCGCACACTAGGTAGAGA
*Pdx1*	ATCTCCCCATACGAAGTGCC	CGTGAGCTTTGGTGGATTTCAT
*Nkx6.1*	GGACTGCCACGCTTTAGCA	TGGGTCTCGTGTGTTTTCTCT
*C/EBPα*	CTTCAGCCCGTACCTGGAG	GGAGAGGAAGTCGTGGTGC
*PPARγ*	GGGATCAGCTCCGTGGATCT	TGCACTTTGGTACTCTTGAAGTT

**Table 2:** Primers for RT-PCR reactions

Gene name	Forward primer	Reverse primer
*β-actin*	AGAGCTACGAGCTGCCTGAC	AGCACTGTGTTGGCGTACAG
*PPARγ*	TTCAGCAGCGTGTTCGACTT	AGGAATCGCTTTCTGGGTCA
*C/EBPα*	CTAACTCCCCCATGGAGTCGG	GTCGATGGACGTCTCGTGC
*CollagenI*	GATGGATTCCAGTTCGAGTATG	GTTTGGGTTGCTTGTCTG TTTG
*CbfaI*	GATGACACTGCCACCTCTGA	GACTGGCGGGGTGTAAGT
*Osteocalcin*	ATGAGAGCCCTCACACTCCTC	CGTAGAAGCGCCGATAGGC
*Osterix*	TAATGGGCTCCTTTCACCTG	CACTGGGCAGACAGTCAGAA
*Bone Sialo protein*	TCAGCATTTTGGGAATGGCC	GAGGTTGTTGTCTTCGAGGT
*Osteonectin*	AGTAGGGCCTGGATCTTCTT	CTGCTTCTCAGTCAGAAGG
*Nestin*	AGCTGGCGCACCTCAAGATG	AGGGAAGTTGGGCTCAGGAC
*GFAP*	GAGGCGGCCAGTTATCAGGA	GTTCTCCTCGCCCTCTAGCA

### Whole genome bisulfite sequencing and bioinformatics analysis

Genomic DNA was extracted from 5000 CD24^+^ or fibroblast cells using the SDS/Proteinase K/Phenol chloroform method [15]. Then 20 pg of unmethylated lambda DNA was spiked into 20 ng of iPMSC genomic DNA. The combined DNA was sheared to 200 bp fragments using a Covaris sonicator. Fragmented DNAs were end-repaired, dA-tailed, and ligated to a methylated NEB Illumina adaptor using the NEBNext® Ultra™ DNA Library Prep Kit for Illumina (E7370S; NEB). Adaptor ligated DNA was bisulfite converted using the EZ DNA Methylation Kit (Zymo Research). Libraries were enriched by PCR using a mutated version of Q5 polymerase that can read DNA templates containing dU (New England Biolabs) and sequenced on the Illumina NextSeq 500 platform with 150 bp paired-end reads. Libraries were made in duplicate. Two high-output runs were carried out.

Adaptor trimmed sequencing reads were mapped to hg19 and bacterial phage lambda genomic sequences and CpG methylation levels were extracted using Bismark [16]. CpG sites with at least two reads covered in two replicate libraries were included in the downstream analysis. CpG methylation levels in genomic regions were obtained by the local likelihood smoothing function in the bsseq R package [17]. Differential methylation between CD24^+^ and fibroblast cells was analyzed using *t* statistics. Regions with a mean differential methylation level higher than 0.1 were defined as differentially methylated regions (DMRs).

DMRs were annotated to the UCSC RefGene table, and their distribution in genomic elements including promoter, 5′ UTR, exon, intron, intergenic, 3′ UTR, TTS, and repetitive sequences was calculated using HOMER [18]. Fold enrichment of DMRs in specific genomic elements and the distance of DMRs to their closest TSS were also calculated using HOMER. Then 60,000 genomic regions, each with a length of 200 bp, were randomly sampled three times from the human genome hg19, and these three random datasets were used as controls when analyzing the distance of DMRs to TSS.

Genes harboring at least one DMR (DMR genes) within the ±2 kb region of TSS were used for gene-set enrichment analysis, since DNA methylation changes located in the TSS or flanking regions are more likely to affect gene expression. A total of 866 genes were investigated for enrichment within the MSigDB database (gene set CGP: chemical and genetic perturbations) using a hypergeometric distribution model and *p* values were corrected to FDR (*q* value) with the Benjamini-Hochberg method [19]. Interaction networks of genes enriched in each gene set were constructed using GeneMANIA [20].

### Directed in-vitro differentiation

The pluripotency of stem cells was examined by directed in-vitro differentiation. For adipogenic differentiation (mesoderm), stem cells were induced in the medium (10 % FBS/DMEM, 500 μM IBMX, 1 μM dexamethasone, 10 μg/ml insulin, and 200 μM indomethacin) for 3 weeks, with media changed every third day. For osteogenic differentiation (mesoderm), iPMSCs were induced in the medium (10 % FBS/DMEM, 50 μM l-ascorbic acid, 10 mM β-glycerophosphate, and 0.1 μM dexamethasone) for 2 weeks, with media changed every third day. For neurogenic differentiation (ectoderm), iPMSCs were first preincubated in 20 % FBS/DMEM, 1 mM β-mercaptoethanol (BME), and 10 ng/ml basic fibroblast growth factor (bFGF; Invitrogen) for 24 h, and then induced in 2 μM valproic acid, 10 μM forskolin, 1 μM hydrocortisone, and 5 μg/ml insulin for 1 week, with media changed every third day. For pancreatic islet differentiation [21–23]: day 1, RPMI (without FBS), activin A (100 ng/ml), and Wnt3a (25 ng/ml); days 2–3, RPMI with 0.2 % vol/vol FBS and activin A (100 ng/ml); days 4–6, RPMI with 2 % vol/vol FBS and FGF-10 (50 ng/ml); days 7–9, DMEM with 1 % vol/vol B27 supplement, cyclopamine (0.25 μM), all-trans retinoic acid (RA, 2 μM), and Noggin (50 ng/ml); and days 10–12, DMEM with 1 % vol/vol B27 supplement.

### Immunofluorescence assay, PCR, and western blotting of differentiated iPMSCs

Induced osteogenic cells were fixed by 4 % paraformaldehyde (Sigma-Aldrich) and stained by anti-osteocalcin antibody (1:200 dilution; Santa Cruz); induced neurogenic cells were stained with anti-GFAP antibody (1:300 dilution; CST), anti-Nestin antibody (1:300 dilution; CST), anti-MAP2 antibody (1:200 dilution; Abcam), and anti-β-Tubulin III (1:200 dilution; Abcam); and induced pancreatic islet cells were stained with anti-Pdx1 antibody (1:400 dilution; CST), anti-glucagon antibody (1:400 dilution; CST), and anti-insulin antibody (1:400 dilution, 3014; CST) respectively. Total RNA was extracted using TRIZol® Reagent (Life Technologies) followed by cDNA synthesis using M-MuLV Reverse Transcriptase and Oligo (dT)23VN (NEB). qPCR was performed with iTaq™ Universal SYBR® Green Supermix (Bio Rad). Western blotting was performed for osteocalcin, microtubule-associated protein 2 (MAP2), β-Tubulin III, ADFP, and Plascidin L. All antibodies were validated for antigen specificity.

### Glucose-stimulated insulin secretion assay

To determine whether cells could respond to glucose in vitro [24], the differentiated cells were preincubated for 4 h at 37 °C in Krebs–Ringer bicarbonate HEPES (KRBH) buffer of the following composition: 129 mM NaCl, 5 mM NaHCO_3_, 4.8 mM KCl, 1.2 mM KH_2_PO_4_, 1.2 mM MgSO_4_, 2.5 mM CaCl_2_, 10 mM HEPES, and 0.1 % (wt/vol) BSA at pH 7.4.

For high glucose-induced insulin release, cells were incubated in KRBH buffer supplemented with different concentrations of glucose (3.3 mM and 16.7 mM) together with 10 μM tolbutamide (Sigma) for 2 h at 37 °C. The concentration of insulin secreted into the culture media was measured using a human insulin enzyme-linked immunosorbent assay (ELISA) kit (CSB-E05069h; CUSABIO).

### Directed in-vivo differentiation on scaffold

Adipogenic iPMSCs and polyglycolic (PGA) fibro scaffold contracts were implanted subcutaneously on the backs of nude mice. Osteogenic iPMSCs and porous β-tricalcium phosphate (β-TCP) scaffold contracts were also implanted subcutaneously on the backs of nude mice. Eight weeks after implantation animals were sacrificed, and the cell scaffold constructs were harvested and fixed in freshly prepared 4 % paraformaldehyde in PBS.

### Histochemistry and immunohistochemistry assay

Following paraformaldehyde fixation, the scaffolds were embedded in paraffin. Ten micron tissue sections were cut using RM2235 radial microtomes (Leica Microsystems) for histological analysis and stained with hematoxylin and eosin (H & E). In the osteogenic group, tissue sections were also stained by a modified Masson Trichrome [25] and Von Kussa [26] staining. Five mice from each group were analyzed. Immunohistochemistry staining of HLA-ABC (CST) at 1:1000 dilution were used to detect whether the tissue formed in vivo originated from human iPMSCs. Sections were observed under an AX80 microscope (Olympus, Tokyo, Japan).

### Teratoma formation

Reprogrammed iPMSC suspensions (1 × 10^7^) were mixed with matrigel (BD Bioscience) and injected subcutaneously into NOD/SCID mice without anesthesia. After 2 months teratomas were collected and fixed in 4 % paraformaldehyde in PBS, embedded in paraffin, and sectioned at 10 μm using RM2235 radial microtomes (Leica Microsystems). Sections were subjected to histologic staining with H & E and immunohistochemistry staining of HLA-ABC as already described and observed under an AX80 microscope (Olympus).

## Results

### Generation and localization of transfected proteins

The purified Oct4, Sox2, c-Myc, and Klf4 were used for HVJ-E packaging (Fig. 1a, b). To determine the intracellular localization of transduced transcription factors by HVJ-E, we used anti-His tag antibody and visualized the localization of these His-tagged protein factors using a microscope. Indeed, 24 h after viral transduction, 90 % of the living cells were positive for His-tag transcription factors and they all localized in the nucleus, suggesting that the transcription factors are indeed transported across the nuclear membrane (Fig. 1c). Fluorescence intensity decreased significantly 96 h post transduction, accounting only for about 5 % fluorescent fibroblasts, suggesting a gradual degradation of the His-tagged transcription factors. Therefore we concluded that HVJ-E is an efficient vehicle for protein transduction into fibroblasts, and recombinant transcription factors remain in the nucleus for at least 72 h (Fig. 1c).

### Generation and validation of reprogrammed stem cells

**Fig. 2:**
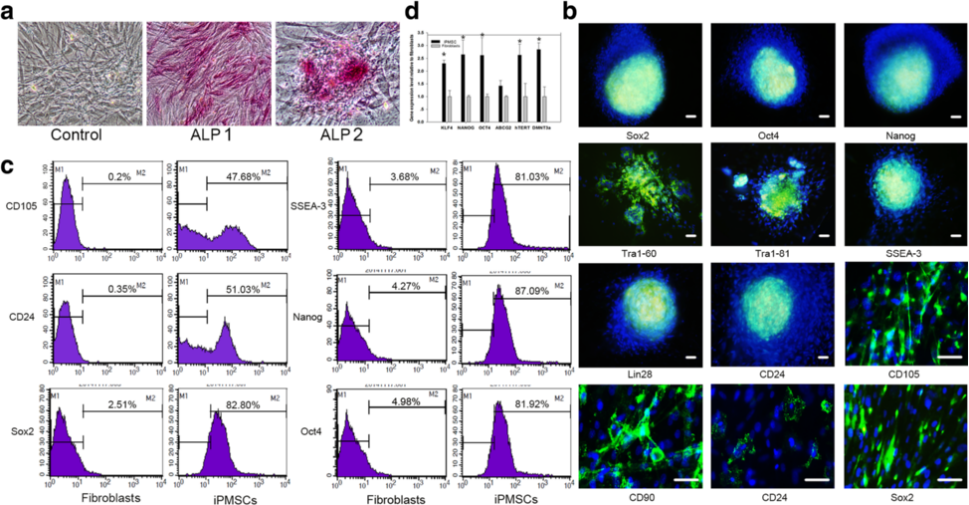
Generation of iPMSCs by HVJ-E transduced purified recombinant transcription factors. **a** ALP staining of putative iPMSCs. Control fibroblasts were without reprogramming and stained with ALP. *ALP1* denotes expanded cells with ALP staining. The expanded iPMSCs grew in compact colonies and stained positive for ALP (*ALP2*). **b** Putative clone expressing hESC markers (*green*), including Sox2, Oct4, Nanog, Lin28, Tra1-60, Tra1-81,SSEA-3, and CD24. Nuclei were stained with Hochest 33342 stain (*blue*) and the images were merged. Scale bars = 50 nM. **c** FACS analysis of the colonies differentiated into fibroblast-shaped cells demonstrating expression of pluripotent stem cell markers including CD105, CD24, and Sox2 (*left*). Surface antigen profiling of the whole population of protein-reprogrammed fibroblasts for Nanog, Oct4, and SSEA-3 is also shown (*right*). **d** Endogenous pluripotency markers of RNA expression in the reprogrammed fibroblasts determined by qPCR. GAPDH was used for normalization. Note that total RNA was extracted from a mixed population of cells with ~30 % displaying stem cell characteristics. *p < 0.01 between control fibroblasts before and after reprogramming using the protein factors. *IPMSC* induced pluripotent mesenchymal stem cell

To generate iPSCs from human fibroblasts, the fibroblasts were subjected to three protein transduction cycles at 72-h intervals (Fig. 1b), as described in Methods. On day 10, the cells were gently dissociated and transferred into VTN-coated 10-cm dishes and cultured until stem cell-like colonies appeared (around day 30). The cells lost their characteristic fibroblast morphology and formed tight, round colonies, with enlarged nuclei and a high nucleus-to-cytoplasm ratio. To verify the undifferentiated state of the generated putative stem cells, we first assayed alkaline phosphatase (ALP) activity. All colonies exhibited positive staining for ALP. Among the reprogrammed fibroblasts that did not form colonies, about 60 % tested were positive for ALP (ALP1 and ALP2; Fig. 2a). To further evaluate the undifferentiated state we performed immuncytochemical staining for the major pluripotency markers Sox2, Oct4, Nanog, Tra1-60, Tra1-81, SSEA-3, CD105, Lin28, and CD24 (Fig. 2b). This staining confirmed uniform expression of these pluripotency markers in all colonies. Based on ALP staining and immuncytochemical staining for the major pluripotency markers, the reprogramming efficiency was about ~30 %.

To establish stable iPSC lines, colonies were seeded on VTN-coated plates and cultured in Essential 8™ medium. Over 7 days of culture, these colonies did not grow, but did differentiate into fibroblast-shaped cells suggesting iPMSCs. Cells additionally stained positive for pluripotency markers CD105, CD90, CD24, and Sox2. We also performed flow cytometry analysis of the whole population of reprogrammed fibroblasts utilizing an extended set of pluripotency markers, namely Nanog, Oct4, Sox2, SSEA-3, CD24, and CD105. Approximately 80 % of the cells were positive for Nanog, Oct4, Sox2, and SSEA-3, and about 50 % of the cells were positive for CD24 and CD105 (Fig. 2c). The relative expression of the pluripotency marker genes was determined for the reprogrammed fibroblast population using qPCR, with housekeeping gene GAPDH as a reference. The fibroblast population was treated with three protein transduction cycles at 72-h intervals and contained a mixed population of reprogrammed cells and fibroblasts. The endogenous pluripotency marker expression levels increased significantly, except ABCG2, compared with fibroblasts (Fig. 2d, Table 1), suggesting the reprogrammed stem cells display all features specific to iPMSC cells, specifically morphology and pluripotency marker expression.

### Epigenome reprogramming of the iPMSCs derived from human fibroblasts

DNA methylation change, especially CpG methylation, is an important epigenetic mark affecting gene expression during the reprogramming process. To help explain expression changes in pluripotency-related genes after introduction of recombinant Yamanaka factors into fibroblasts, and to investigate the epigenetic mechanisms underlying reprogramming, we performed whole genome bisulfite sequencing on the CD24^+^ iPMSCs and corresponding control fibroblast cells.

A total number of 310 million WGBS reads from the CD24^+^ libraries were mapped to hg19 and 370 million reads from the fibroblast libraries were mapped, representing about 10× genome coverage for each individual library. Approximately 44 million CpG sites were covered by each library, estimated to be 78.6 % of all CpG sites in the human genome. Also, using lambda DNA spike-in controls, we calculated that the bisulfite conversion rate of each library was greater than 99 %.

**Fig. 3:**
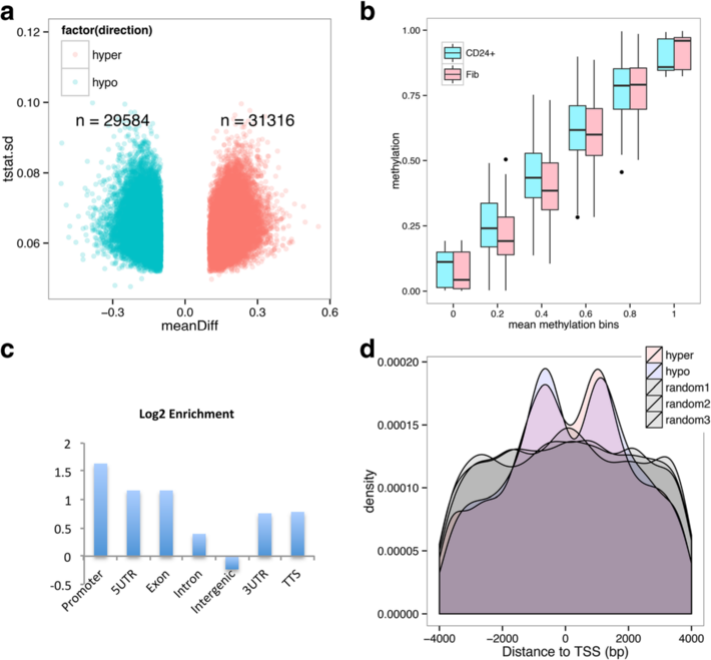
WGBS revealed CpG methylation alterations in iPMSCs compared with fibroblast cells. **a** Volcano plot depicting the DMRs in iPMSCs compared with fibroblasts (*Fib*), with the horizontal axis representing methylation differences and the vertical axis representing the standard deviation (*sd*) in *t* statistics. **b** Box plot showing the distribution of CpG sites based on different methylation levels. **c** Bar plot showing the enrichment of DMRs in different genomic features. **d** Distribution of DMR distances to its closest TSS; controls are generated by randomly sampling 60,000 regions of 200 bp from hg19

We analyzed the differential methylation between CD24^+^ and fibroblast cells using a smoothing coupled *t*-statistics approach. By setting a differential methylation threshold of 0.1 (10 %), 29,584 hypomethylated DMRs and 31,316 hypermethylated DMRs were identified (Fig. 3a). While the numerical distribution of CpG sites in each DMR and the length of DMRs are similar between the hypomethylated and hypermethylated groups (Additional file 2: Figure S2A, B), there is a difference in the distribution of DMRs across CpG methylation levels. Specifically, we found that the gain of methylation (hypermethylated DMRs) occurs in regions with low-to-medium extents of methylation, while the loss of methylation (hypomethylated DMRs) occurs in regions that were originally highly methylated (Fig. 3b).

Genomic annotation revealed that DMRs were enriched in functional elements (i.e.*,* promoter, 5′ UTR, exon, 3′ UTR, and TTS), most especially in promoter regions (highest log_2_-fold enrichment, Fig. 3c), but depleted in intergenic regions, which play a less functional role in gene expression (Fig. 3c). No apparent DMR enrichment was found in repetitive elements (data not shown). Since DMRs are mostly enriched in promoter regions, we analyzed the distribution of the distances of each DMR to its closest TSS. Remarkably, distance density plots demonstrated that both hypermethylated and hypomethylated regions extend from TSS ± 1 kb regions with a bi-peak distribution pattern and are depleted at TSS sites, but not at three random region controls (Fig. 3d and Additional file 2: Figure S2C, D), indicating that introduction of the four recombinant transcription factors leads to changes in the DNA methylation patterns of promoter regions, possibly correlated with gene expression. The depletion of a DMR at its TSS, however, could be due to the occupancy of H3K4me3 and H2A.Z histone variants at the TSS, which are strongly antagonistic to DNA methyltransferase targeting (DNMT) and thus keep TSS persistently hypomethylated [27].

A functional study was carried out using genes with at least one DMR in their promoter region. Gene set enrichment analysis revealed that DMR genes are highly enriched in stem cell-related categories (Table 3). Besides enrichment in general stem cell genes, genes with DMR were also observed in sets that are regulated by stem cell-related transcription factors (KLF1, MAPK8, NANOG, SUZ12, SOX2, and MYC). These enrichment results indicate that the transduced recombinant transcription factors regulate the programmed DNA methylation of stem cell-related genes.

**Table 3:** Gene set enrichment analysis of genes with at least one DMR in the promoter

Gene set name	Number of genes in gene set (*K*)	Number of genes in overlap (*k*)	*k*/*K*	False Discovery Rate *q* value
PILON_KLF1_TARGETS_DN	1972	107	0.0543	2.01 × 10^–19^
YOSHIMURA_MAPK8_TARGETS_UP	1305	73	0.0559	5.87 × 10^–14^
BOQUEST_STEM_CELL_UP	260	31	0.1192	1.14 × 10^–13^
BENPORATH_NANOG_TARGETS	988	61	0.0617	1.70 × 10^–13^
LIM_MAMMARY_STEM_CELL_UP	489	40	0.0818	1.73 × 10^–12^
WONG_ADULT_TISSUE_STEM_MODULE	721	46	0.0638	9.24 × 10^–11^
PASINI_SUZ12_TARGETS_DN	315	28	0.0889	9.54 × 10^–10^
BENPORATH_SOX2_TARGETS	734	43	0.0586	4.40 × 10^–9^
BENPORATH_ES_WITH_H3K27ME3	1118	53	0.0474	5.27 × 10^–8^
ZHANG_TLX_TARGETS_60HR_UP	293	24	0.0819	9.01 × 10^–8^
DANG_BOUND_BY_MYC	1103	51	0.0462	2.09 × 10^–7^

*DMR* differentially methylated region

To further understand the mechanism of DNA methylation changes exerted by Yamanaka transcription factors, we constructed gene interaction networks using the DMR genes enriched in specific categories (Table 3). Indeed, enriched genes could be readily organized into networks, and Yamanaka transcription factors showed extensive interaction relationships with enriched gene nodes, indicating a legitimate relationship between altered DNA methylation and introduction of Yamanaka transcription factors (Additional file 3: Figure S3).

An overlapping analysis of enriched DMR genes in different gene sets revealed that FEZ1 and OLFML3 were found in STEM_CELL_UP, NANOG_TARGETS, and SOX2_TARGETS sets. FEZ1 is essential for normal axonal bundling and elongation within axon bundles and OLFML3 plays an essential role in dorsoventral patterning during early development. The DNA methylation change in these genes is also consistent with the gain of pluripotency following transduction of Yamanaka transcription factors into human fibroblasts (Additional file 4: Figure S4).

### In-vitro differentiation of iPMSCs

**Fig. 4:**
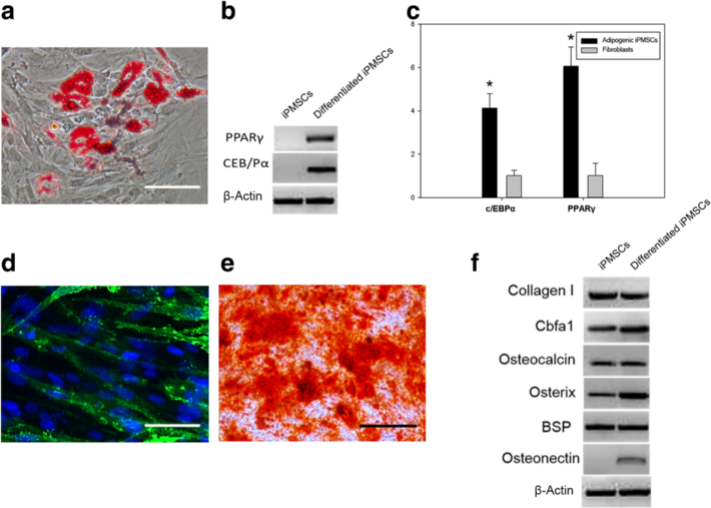
In-vitro adipogenic and osteogenic differentiation potential of induced stem cells. **a** iPMSCs were induced into adipocytes in defined medium and lipid droplets were subsequently stained by Oil Red O. **b** mRNA expression levels of PPARγ and C/EBPα marker genes in the adipogenic group using RT-PCR and resolving the amplicon on agarose gel. **c** Expression levels of PPARγ and C/EBPα compared with control group using qPCR. Note that total RNA was extracted from mixed population of cells with ~30 % displaying stem cell characteristics. *p < 0.01 between control IPMSCs before differentiation (fibroblasts) and after adipogenic conversion (adipogenic IPMSCs). **d** iPMSCs were induced into osteoblasts and subsequently stained for osteocalcin. **e** Alizarin Red staining identified the calcium nodules secreted by induced osteoblasts. **f** Osteoblast specific gene expression in iPMSCs and the osteogenic group by RT-PCR and subsequently resolving the amplicon on agarose gel. *IPMSC* induced pluripotent mesenchymal stem cell

The main feature of the stem cell is its inherent property to differentiate into different cell types. Characteristic stem cell properties can be assayed both in vitro and in vivo. In-vitro differentiation assays were performed to assess the developmental potential of the iPMSCs derived by protein factors. iPMSCs were induced into adipocytes in medium containing 1 μM dexamethasone, 200 μM indomethacin, 500 μM IBMX, and 10 μg/ml insulin for 2 weeks. Oil Red droplets were observed in >50 % of the iPMSCs, demonstrating an efficient differentiation process of the cells (Fig. 4a). The mRNA expression of important transcription factors involved in adipogenesis, such as PPARγ and C/EBPα, was also significantly increased by RT-PCR (Fig. 4b) and qPCR assay between fibroblast-derived control iPMSCs and adipogenic iPMSCs (Fig. 4c). We also analyzed the osteogenic differentiation potential of the iPMSCs. After being induced into osteoblasts with 50 μM l-ascorbic acid, 10 mM β-glycerophosphate, and 0.1 μM dexamethasone for 2 weeks, the cells were stained for osteocalcin and Hoechst 33342. Indeed, osteocalcin was deposited throughout except in the nucleus (Fig. 4d). Furthermore, Alizarin Red staining displayed calcium nodules secreted by induced osteoblasts in 90 % of the cells (Fig. 4e). We also performed RT-PCR analysis to determine gene expression for collagen I, cbfa1, osteocalcin, osterix, BSP, and osteonectin. Indeed, only osteogenic-specific genes cbfa1, osterix, and osteonectin were found to be overexpressed following differentiation compared with parental iPMSCs (Fig. 4f).

**Fig. 5:**
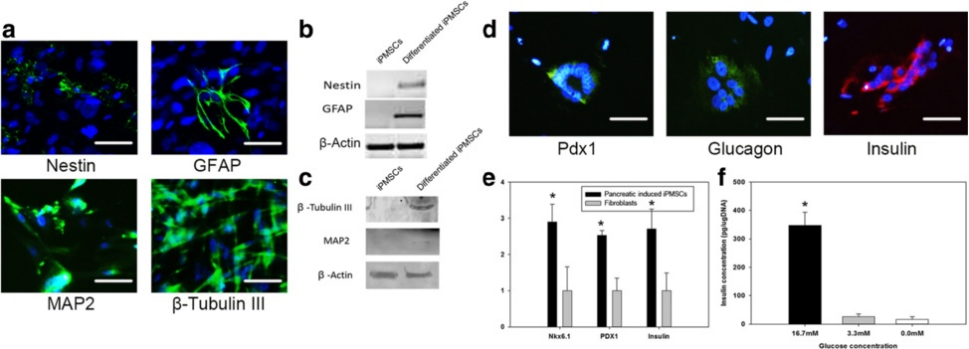
In-vitro neurogenic and pancreatic differentiation potential of induced stem cells. iPMSCs can be induced into neuron by defined media. **a** Induced cells showed typical neuron morphology with soma, dendrites, and axons. GFAP, Nestin, MAP2, and β-Tubulin III marker proteins were identified by immunofluorescence staining. **b** GFAP, Nestin, MAP2, and β-Tubulin III in protein expression in differentiated iPMSCs. **c** iPMSCs were induced into islet cells. **d** Typical endocrine aggregates and expressed pancreatic specific markers such as Pdx1, glucagon and insulin are shown. Pdx1 was detected by anti-Pdx1 antibody whereas glucagon and insulin were stained. **e** Gene expression of Pdx1, glucagon, and insulin in induced cells (pancreatic iPMSCs) and control group before induction (fibroblasts) (*left panel*). Note that total RNA was extracted from a mixed population of cells with ~30 % displaying stem cell characteristics. *p < 0.01 between control IPMSCs before differentiation (fibroblasts) and after pancreatic conversion (pancreatic induced IPMSCs). **f** Insulin secretion from induced pancreatic cells in response to various concentration of glucose. Bars represent an average of three reads. *p < 0.01 compared with 3.3 mM glucose induction. *GFAP* glial fibrillary acidic protein, *IPMSC* induced pluripotent mesenchymal stem cell, *MAP2* microtubule-associated protein 2, *Pdx1* pancreatic and duodenal homeobox 1

Similarly, neural differentiation iPMSCs were replanted on a poly-d-lysin/laminin-coated surface and first induced in DMEM culture medium containing 20 % FBS, 1 mM BME, and 10 ng/ml bFGF for 24 h. Media were then changed to DMEM containing 10 % FBS supplemented with 5 mM KCl, 2 μM VPA, 10 μM forskolin, 1 μM hydrocortisone, and 5 μg/ml insulin and cultures were maintained for 14 days. Seven days after induction, cells started to develop a neuronal morphology, namely neural cells with neuraxon and dendrites were observed by light microscopy. The cells were additionally positive for nestin (a neural progenitor cell (NPC) marker), for glial fibrillary acidic protein (GFAP; an intermediate filament (IF) protein that is expressed by numerous cell types of the central nervous system (CNS)), for MAP2 (a neuron-specific cytoskeletal protein), and for neural class III β-Tubulin (β-Tubulin III; a neuron marker) (Fig. 5a). These observations demonstrated that iPMSCs were able to generate NPCs and differentiate further into mature neurons. RT-PCR analysis detected nestin and GFAP expression at the early stages of NPC formation (Fig. 5b), followed by MAP2 and β-Tubulin III expression as cells differentiated further into mature neurons 14 days post induction (Fig. 5c).

In yet another example, iPMSCs were induced into islet cells over a 12-day period. After induction, typical endocrine aggregates were formed. Immunofluorescent staining showed that the cell clusters were positive for insulin. It has been reported that the immunoreactivity of insulin in the cell clusters might be due to the uptake of insulin from the media. To distinguish this possibility and to characterize pancreatic differentiation in detail, immunofluorescent staining of pancreatic endocrine hormones was performed, including glucagon, which is synthesized and secreted from alpha cells of the islets (Fig. 5d). Pancreatic and duodenal homeobox 1 (Pdx1), a transcription factor necessary for pancreatic development and β-cell maturation, also stained positively in induced iPMSCs (Fig. 5d). The gene expression profile analyzed by qPCR further confirmed the pancreatic differentiation of iPMSCs, as the relative gene expression levels of insulin, glucagon, and Pdx1 were significantly increased compared with the original control fibroblast-derived iPMSCs (Fig. 5e). To determine whether the differentiated iPMSCs were responsive to glucose challenge, insulin release after exposure to high glucose was measured using a human insulin ELISA kit. Insulin secretion after cells were exposed to high concentrations of glucose (16.7 mM) increased significantly compared with cells exposed to low concentrations of glucose (3.3 mM), as well as KRBH buffer without glucose (Fig. 5f).

### In-vivo differentiation of iPMSCs

**Fig. 6:**
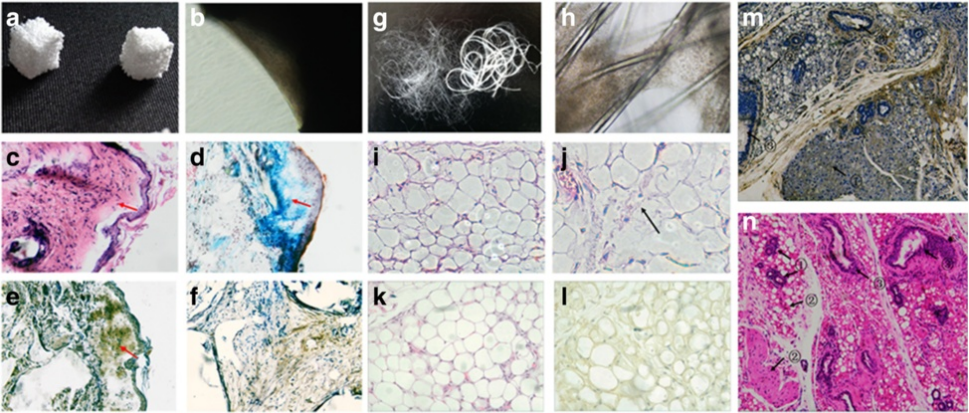
In-vitro differentiation potential of iPMSCs. **a** β-TCP was used for in-vivo osteogenic differentiation. **b** Differentiated iPMSCs effectively adhered to and proliferated on the scaffold. **c** H & E staining shows the structure of osteogenic tissue formed in vivo 2 months after implantation. **d** Masson Trichrome staining shows the collagen fibers in the tissue. **e** Von Kussa staining confirms the presence of matrix mineralization. **f** HLA-ABC immunohistochemistry staining confirmed the osteogenic tissue originated from human cells. **g** PGA was used for in-vivo adipogenic differentiation. **h** Reprogrammed fibroblasts were seeded on a PGA scaffold and cultured in the adipogenic induction medium for 3 weeks. Induced fibroblasts effectively adhered to and proliferated on the porous PGA scaffold. **i** H & E staining show the structure of adipogenic tissue formed in vivo 2 months after implantation. **j** Lipid vacuoles were observed. *Arrow* indicates the undegraded PGA scaffold in vivo. **k** H & E staining for adipogenic tissue originated from human cells. **l** HLA-ABC immunohistochemistry staining confirmed the adipogenic tissue originated from human cells. **m** HLA-ABC immunohistochemistry staining of the teratomas confirmed it originated from human cells (*①* endoderm, *②* mesoderm, *③* ecoderm). **n** Teratoma formation from subcutaneous injection of iPMSCs into NOD/SCID mice. Tissues from endoderm, mesoderm, and ectoderm were formed in the teratomas (shown by H & E staining, *①* endoderm, *②* mesoderm, *③* ectoderm)

To establish the ability of iPMSCs to differentiate in vivo, reprogrammed fibroblasts were seeded on a porous β-TCP (Fig. 6a) scaffold and cultured in the osteogenic induction medium for 3 weeks. β-TCP showed high biocompatibility for induced fibroblasts as indicated by cell adherence and proliferation on the porous β-TCP scaffold (Fig. 6b). After 3 weeks, β-TCP scaffold constructs were implanted subcutaneously on the backs of nude mice. Two months after implantation the animals were sacrificed, the scaffolds harvested, and histological analysis was performed (Fig. 6c). Masson Trichrome staining identified collagen synthesis (Fig. 6d) and Von Kussa staining revealed the presence of matrix mineralization in newly formed osteogenic tissue (Fig. 6e). HLA is the major histocompatibility complex (MHC) specific to humans. HLA-ABC immunohistochemistry staining demonstrated that the osteogenic tissue originated from human cells (Fig. 6f).

iPMSCs were also seeded onto a PGA (Fig. 6g) scaffold and cultured in adipogenic induction medium for 3 weeks. Induced cells adhered and proliferated on the biocompatible porous PGA scaffold (Fig. 6h). Adipogenic fibroblast scaffold constructs were implanted subcutaneously on the backs of nude mice for 2 months, at which stage cell scaffold constructs were found to have grown into adipose tissues in vivo. H & E staining revealed lipid vacuoles while the undegraded PGA scaffold remnants were found in the adipose tissue (Fig. 6i, j, arrow). HLA-ABC immunohistochemistry staining also confirmed that the adipogenic tissue originated from human cells (Fig. 6k).

### Teratoma formation

Reprogrammed fibroblast cell suspensions (1 × 10^7^ cells) were mixed with matrigel and injected subcutaneously into SCID mice without anesthesia. Teratoma formation was observed after transplantation of iPMSCs subcutaneously on the backs of NOD/SCID mice for 2 months. These teratomas contained tissues from all three germ layers and included neural (ectoderm), epidermal (ectoderm), striated muscle (mesoderm), adipose (mesoderm), and liver (endoderm) tissues, confirming the pluripotency of the iPMSCs both in vitro and in vivo (Fig. 6l). HLA-ABC immunohistochemistry staining confirmed the teratomas were originated from human cells (Fig. 6m). H & E staining shows the teratomas were composed of tissues from three different germ layers (Fig. 6n).

## Discussion

Reprogramming of adult somatic cells into pluripotent stem cells is believed to provide an attractive source of stem cells for basic biological studies and regenerative medicine. This has emerged as a potential method for generating person-specific stem cells of any cell lineage, bypassing the use of ESCs. Various methods are in use to generate iPSCs from somatic cells, most using genome integration by recombinant viral genomes that encode Yamanaka factors, transfection of nucleic acids encoding Yamanaka factors, or introduction of small molecule inducers of iPSCs.

Adult stem cells have been used over years in medicine. MSCs have been evaluated for regenerative medicine applications either through direct differentiation into these tissues or indirectly through protein or cytokine secretion. Patient-derived MSCs have been considered safe because they do not show tumor formation after transplantation and are robust for various differentiations. Concerns over the safety of viral-based technologies have motivated using nucleic acids and small molecule-based technologies to produce autologous stem cells for clinical therapy. However, even these approaches need further testing to determine whether secondary effects will undermine their safe use. Previous use of purified transcription factor for generating iPSCs was successful, although the efficiency of iPSC formation was extremely low, limiting real-life applications. Here we demonstrate improved reprogramming of somatic cells using a different approach from previous methodologies [8, 9, 28]. Three key reprogramming factors (Oct4, Sox2, and c-Myc) were expressed in *Escherichia coli* as inclusion bodies, which were then solubilized, refolded, and further purified in our studies. The fourth reprogramming factor (Klf4) was expressed in HEK293 human cells and was soluble. Our process is therefore capable of producing large quantities of purified recombinant reprogramming factors for clinical applications. Indeed, various solubilization and refolding techniques for processing inclusion body proteins to bioactive proteins have been developed to allow facile and large-scale production of therapeutic proteins [29].

The second improvement of our technology is the reprogramming factor packaging and delivery into the human cells. HVJ-E was originally developed as a vector for the delivery of drugs and genes [30–32]. Furthermore, HVJ-E has also been used for protein transduction to investigate the function and character of proteins, such as enzymes, cytokines, in vivo, or in cells [30, 33, 34]. We found effective delivery by HVJ-E of packaged reprogramming factors to human fibroblasts, with the majority of cells showing positive staining for the exogenous transferred protein at a concentrations of (8 ~ 10) × 10^-3^ μg/ml. We estimated an amount of 1 μg of each protein for 2 × 10^6^ cells to make iPMSCs. Another major improvement over small molecular and nucleic acid introduction is the rapid turnover (>50 %) of the reprogramming factors, estimated to be 72 h post transduction, thus leaving a limited biological footprint on the recipient cell. The protein concentration we used was much lower than reported previously, demonstrating higher efficiency [9]. We also demonstrated that a HVJ-E-based packaging and delivery system can directly reprogram fibroblasts into different cell types such as osteoblasts, adipocytes, pancreatic islets, and neural and teratoma cells.

During development and reprogramming, epigenetic modifiers are thought to play a key role in ensuring the correct patterns of DNA methylation and histone modification for gene expression. Alteration of global epigenetic marks occurs naturally during specific stages in the mammalian life cycle, and can also be artificially engineered using a variety of reprogramming strategies. To validate that HVJ-E packaged transcription factors did indeed alter the epigenome we analyzed the differential methylation between CD24^+^ and fibroblast cells. We observed gain of methylation (hypermethylated DMRs) in low-to-medium methylated regions concurrent with loss of methylation (hypomethylated DMRs) in highly methylated regions. These genes included gene sets that are regulated by stem cell-related transcription factors (KLF1, MAPK8, NANOG, SUZ12, SOX2, and MYC), confirming that transduced recombinant transcription factors regulated the programmed DNA methylation of stem cell-related genes. As reported previously, we also found stem cell-specific DMRs were significantly biased towards CpG islands, suggesting that CpG islands containing genes have a propensity to be differentially methylated during reprogramming towards pluripotent stem cells [35]. The higher number of stem cell-specific DMRs in iPMSCs affecting pluripotency gene expression indicates that the Yamanaka factors only activate limited numbers of stem cell-specific/associated genes through differential methylation compared with the fibroblast genome.

Pluripotent cells derived by reprogramming fibroblast cells could be further differentiated into different cell types. Pluripotent stem cells produced with these recombinant proteins were successfully differentiated into derivatives of all three embryonic germ layers in vitro and in teratomas. Reprogrammed iPMSCs could additionally form osteogenic and adipogenic tissues on scaffolds in vivo. During our transgene-free iPSC reprogramming by protein factors we observed a large number of cells to be positive for CD105, a typical MSC marker, suggesting that the fibroblasts may have undergone quick derivation from iPSCs to MSCs. MSCs derived from human iPSCs can be generated in clonal expansion cultures and can be differentiated into osteoblasts, adipocytes, and chondrocytes and promote vascular and muscle regeneration. More detailed examination is needed to understand this phenomenon.

The field of cell reprogramming is expanding very rapidly, and the tools for safer and more efficient reprogramming have become increasingly important both for basic research and for development of medical applications. Protein-based iPMSC technology offers a new and potentially safer method for generating patient-specific stem cells that does not require the destruction of ex-utero embryos.

## Conclusion

In summary, we describe a noninvasive, transgene-free, protein-mediated approach to reprogramming somatic cells. The reprogramming events led to epigenome alteration and a specific phenotype. This approach eliminates the potential risk associated with the use of viruses, DNA transfection, and harmful chemicals. We anticipate that protein-based reprogramming in future could provide a safe source of patient-specific cells for regenerative medicine.

## Additional files

Additional file 1: Figure S1. Showing isotype control for (**A**) anti-mouse, (**B**) anti-rabbit, and (**C**) anti-rat. (TIF 145 kb) Additional file 2: Figure S2. Showing statistics of DMRs. Distribution of number of CpG sites (**A**) and width (**B**) of DMRs. Distribution of DMR distances to closest TSS (**C**, **D**); controls are generated in the same way as shown in Fig. 3d. (TIF 2435 kb) Additional file 3: Figure S3. Showing interaction network of genes enriched in different stem cell-related gene sets (gene sets are annotated for each network). (TIF 19410 kb) Additional file 4: Figure S4. Showing genomic tracks of OLFML3 and FEZ1, indicating CpG methylation in CD24^+^ cells (*red curves*) and fibroblast cells (*blue curves*). *Pink-shaded* regions are DMRs. (TIF 2321 kb)
