# Supplementary figures and images for: High-efficiency generation of induced pluripotent mesenchymal stem cells from human dermal fibroblasts using recombinant proteins
Source: Stem Cell Res Ther. 2016 Jul 30;7:99. doi: 10.1186/s13287-016-0358-4 (PMC4967313; doi:10.1186/s13287-016-0358-4)

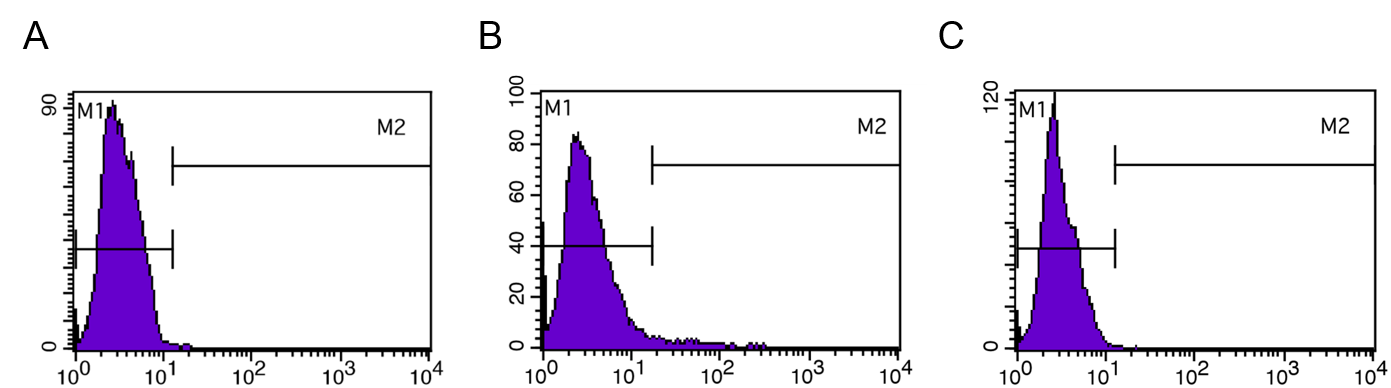

Supplement: Additional file 1: Figure S1. — Showing isotype control for (A) anti-mouse, (B) anti-rabbit, and (C) anti-rat. (TIF 145 kb) [file 13287_2016_358_MOESM1_ESM.tif]

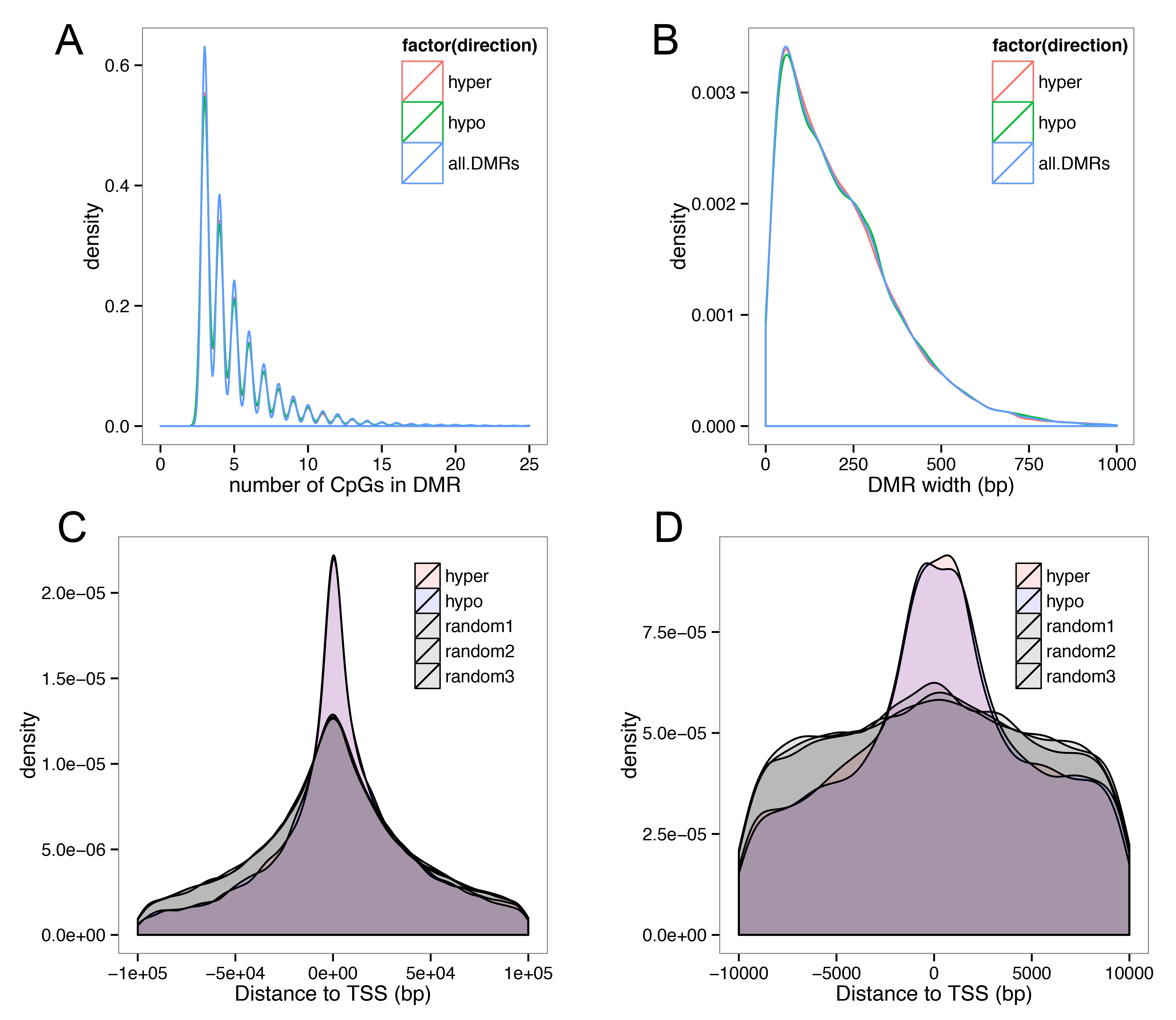

Supplement: Additional file 2: Figure S2. — Showing statistics of DMRs. Distribution of number of CpG sites (A) and width (B) of DMRs. Distribution of DMR distances to closest TSS (C, D); controls are generated in the same way as shown in Fig. 3d. (TIF 2435 kb) [file 13287_2016_358_MOESM2_ESM.tif]

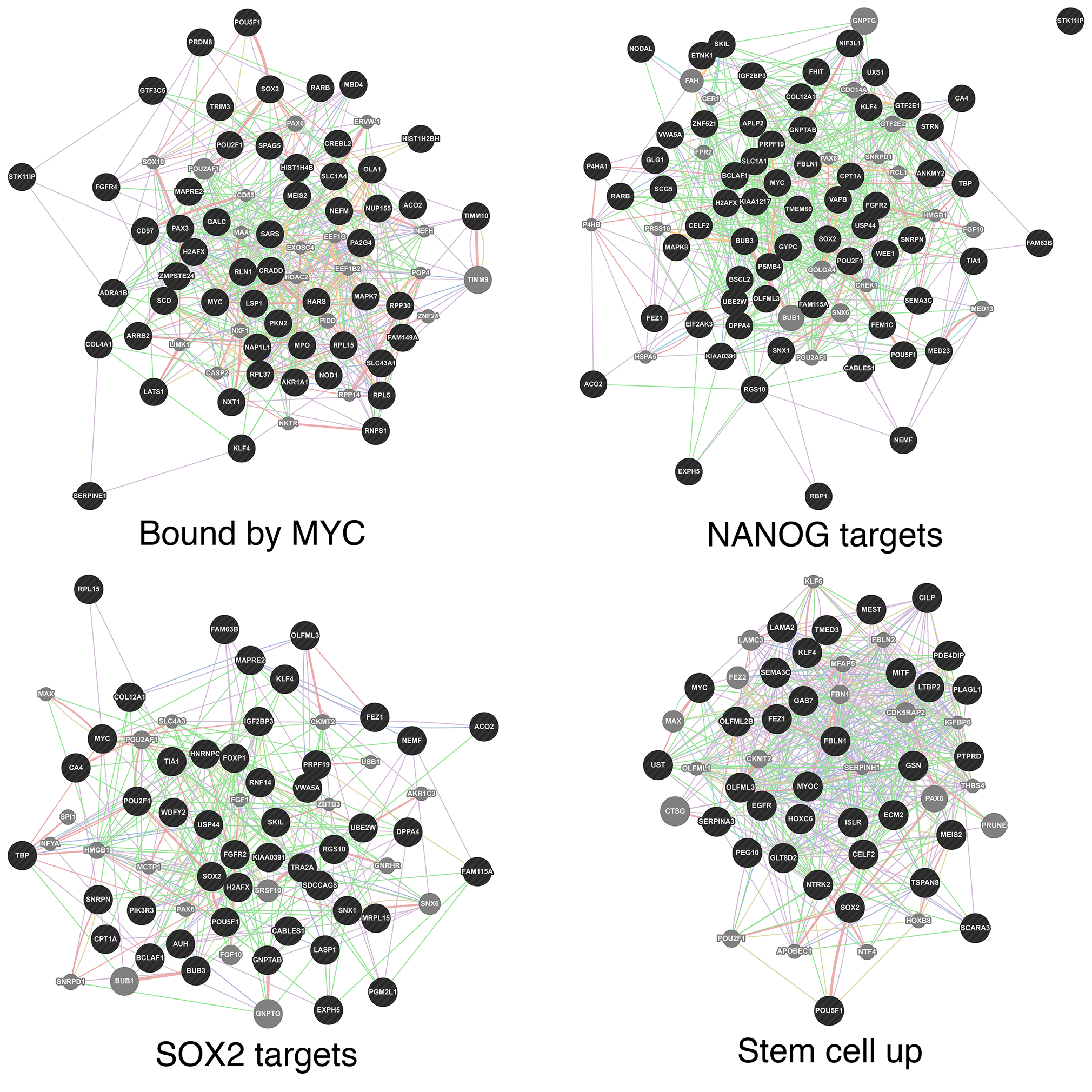

Supplement: Additional file 3: Figure S3. — Showing interaction network of genes enriched in different stem cell-related gene sets (gene sets are annotated for each network). (TIF 19410 kb) [file 13287_2016_358_MOESM3_ESM.tif]

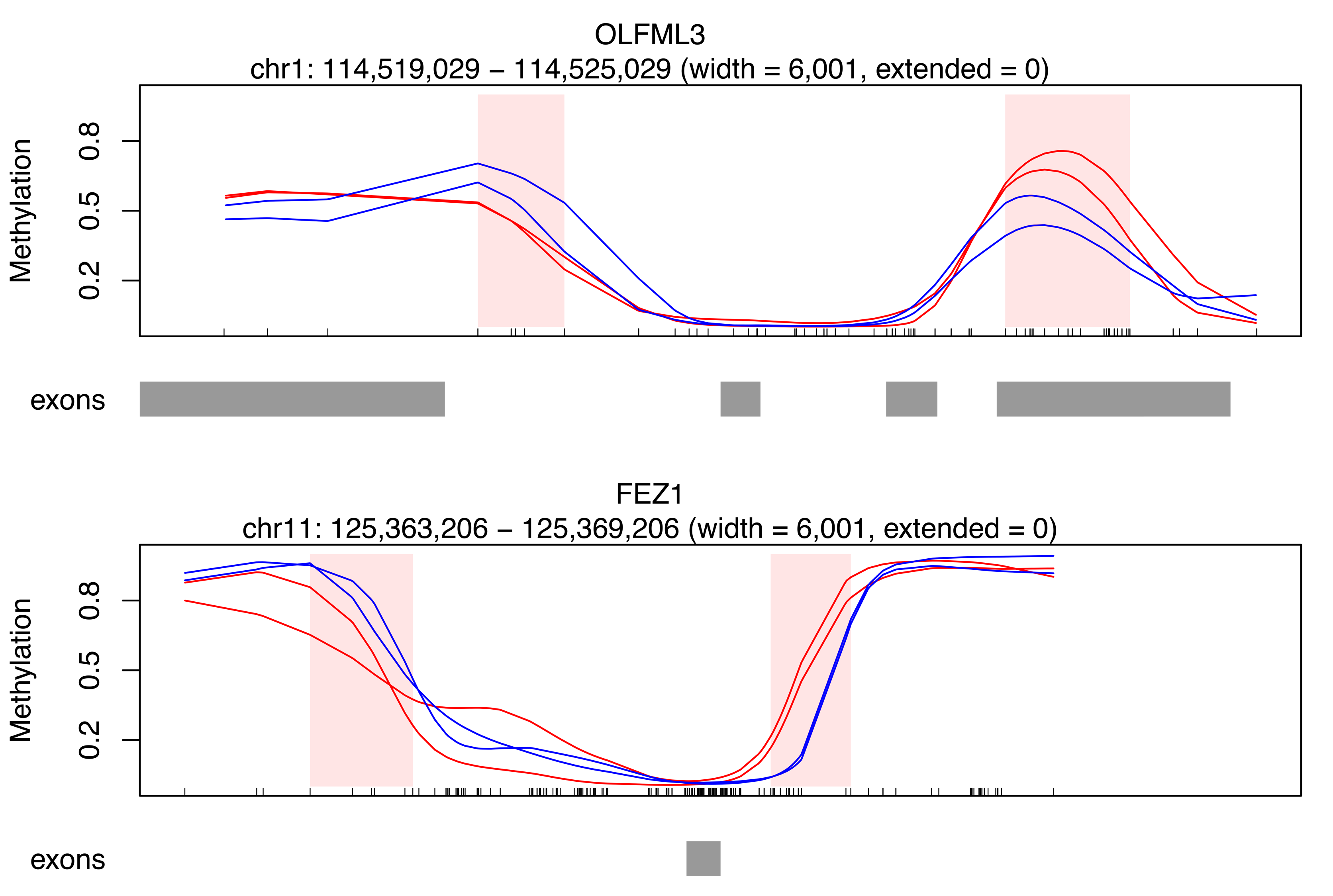

Supplement: Additional file 4: Figure S4. — Showing genomic tracks of OLFML3 and FEZ1, indicating CpG methylation in CD24+ cells (red curves) and fibroblast cells (blue curves). Pink-shaded regions are DMRs. (TIF 2321 kb) [file 13287_2016_358_MOESM4_ESM.tif]
